# Nurses' Self‐Reported Practices and Prescribers' Expectations in Intravenous Fluid Therapy for Hospitalised Patients: A Survey Study and Clinical Documentation Review

**DOI:** 10.1111/jan.70216

**Published:** 2025-09-11

**Authors:** Denise Spoon, Erwin Ista, Myrthe van der Zanden, Monique van Dijk, Elke Berger, Corstiaan den Uil, Jelmer Alsma

**Affiliations:** ^1^ Department of Internal Medicine, Section Nursing Science, Erasmus MC University Medical Center Rotterdam Rotterdam the Netherlands; ^2^ Division of Pediatric Intensive Care, Department of Neonatal and Pediatric Intensive Care Erasmus MC – Sophia Children's Hospital, University Medical Center Rotterdam Rotterdam the Netherlands; ^3^ Franciscus Academy, Franciscus Gasthuis & Vlietland Rotterdam the Netherlands; ^4^ Department of Intensive Care Medicine Maasstad Hospital Rotterdam the Netherlands; ^5^ Department of Intensive Care Medicine Van Weel‐Bethesda Dirksland the Netherlands; ^6^ Department of Intensive Care Medicine, Erasmus MC University Medical Center Rotterdam Rotterdam the Netherlands; ^7^ Department of Internal Medicine, Erasmus MC University Medical Center Rotterdam Rotterdam the Netherlands

**Keywords:** hospital, intravenous fluid therapy, low value care, nurses/nursing care

## Abstract

**Aims:**

To assess self‐reported practices and knowledge of nurses and prescribers (i.e., physicians and nurse practitioners) on intravenous fluid therapy, and to evaluate how this is documented through a clinical documentation review.

**Design:**

Multicentre cross‐sectional study, between April 2022 and July 2022, across 13 wards from four Dutch hospitals.

**Methods:**

A survey study was conducted to assess self‐reported practices related to intravenous fluid therapy. A 12‐item questionnaire evaluated knowledge. To gain insights into documentation practices, a retrospective chart review was performed. Data analysis involved descriptive statistics, with group differences analysed using the chi‐squared test or Fisher's exact test, as appropriate.

**Results:**

Three hundred and four healthcare professionals completed the questionnaire (92% nurses). The majority of prescribers (*n* = 20/25; 80%) expected that nurses would start, stop or change intravenous fluid therapy. Overall, the median number of correct answers to knowledge questions was eight (IQR 7–9, range 0–12); four participants (1%) answered all knowledge questions correctly. Knowledge about the composition of sodium chloride 0.9% solution was limited. Analysis of patient charts revealed that 54% (196/362) received intravenous fluids, most commonly 0.9% sodium chloride infusion (168/195; 86%), although the indication was described in 3% (6/196). Thirty‐one percent (61/196) of patients received intravenous fluids to keep the vein open (< 30 mL/h).

**Conclusion:**

The study identified shared responsibility, a knowledge gap, and limited documentation concerning intravenous fluids. Prescribers expect nurses to adjust intravenous fluids without consulting a prescriber, which aligns with what nurses do, although they are not legally authorised. Given the limited documentation of the indication for intravenous fluids, it is plausible that several patients received intravenous fluids unnecessarily.

**Implications:**

The perceived shared responsibility presents an opportunity to develop a protocol engaging both prescribers and nurses, aiming to guide more targeted infusion therapy.

**Impact:**

Reducing unnecessary infusions to keep‐the‐vein‐open can help eliminate low‐value care.

**Reporting Method:**

CROSS guideline.

**Patient or Public Contribution:**

No patient or public contribution.

**Summary**

- What does this paper contribute to the wider global clinical community?
  ○ Highlights the knowledge gap of healthcare providers on general wards in hospitals regarding intravenous fluid therapy.
  ○ Provides insight into the documentation of the indications for intravenous fluid therapy, highlighting that it is not considered medication in practice.
  ○ Reflects the blind spot among the participants about the importance of sufficient knowledge and awareness of the risks associated with intravenous fluid therapy.

## 1 Introduction

It is estimated that 80% of hospitalised patients receive intravenous fluids (Doyle and McCutcheon 2015; Gawronska et al. 2022; Hoste et al. 2014). A recent point prevalence study in Belgium found that intravenous fluids were administered to 60% of admitted patients (Sneyers et al. 2025). In general, intravenous fluids are indicated for patients whose needs cannot be met by oral or enteral routes, and should be stopped as soon as possible (National Institute for Health and Care Excellence [NICE] 2017). Justifiable indications are volume resuscitation in cases of hypovolemia, sepsis, and perioperative volume losses (Davison et al. 2014; Hoste et al. 2014), and the need to enhance circulating volume to optimise oxygen delivery to tissues (Davison et al. 2014; Navarro et al. 2015). In some cases, indications such as postoperative nausea and vomiting (Chaudhary et al. 2008), metabolic correction (Hoorn 2017), and keeping the vein open (NICE 2017; Paquet and Marchionni 2016) are justifiable as well. Although supporting evidence for the latter indication is lacking, this practice is still common (Doyle et al. 2021).

Intravenous fluid therapy is not without risk, as administering inappropriate amounts or the wrong types of fluid can lead to severe complications (Doyle and McCutcheon 2015), such as fluid overload and acid–base disorders (Kilic et al. 2020). Additionally, complications can occur at the venous access point, including local or systemic infections, thrombophlebitis, infiltration, and extravasation (Chaudhary et al. 2020). Documentation and prescription errors are also highly prevalent (Imogen et al. 2018; Mousavi et al. 2012).

To prevent complications, it is paramount that prescribers (physicians or nurse practitioners) of intravenous fluid therapy have sufficient knowledge about the physiologic principles that determine volume distribution (Davison et al. 2014). However, several studies indicate that both physicians and nurses lack this knowledge (Sindahl et al. 2021; Tailor et al. 2020; Wuyts et al. 2022), and new graduate physicians have expressed concerns that their training did not adequately prepare them for prescribing intravenous fluids (McCrory et al. 2017).

Another crucial aspect of intravenous infusion therapy is the appropriate documentation of its features, including the type of fluid, rate, and duration in patient charts. Inadequate documentation limits the possibility to appropriately review and adjust ongoing intravenous fluid therapy (NICE 2017). The NICE demonstrated that documentation often lacks indications for intravenous fluids, as well as 24‐h plans, documentation of patient weight or requests for further weights, and current or requested fluid balance charts (Sansom and Duggleby 2014).

## 2 Background

Dutch regulations prohibit registered nurses from starting, changing or stopping medication, including intravenous fluids, without a prescription (‘Healthcare Professionals Act [in Dutch: Wet BIG]’; Overheid.nl 1993; de Huisman‐Waal et al. 2019). Registered nurses in the Netherlands are permitted to carry out certain reserved procedures that fall within their area of expertise only when there is a formal instruction from an independently authorised healthcare provider. These procedures are performed without the need for supervision or direct involvement from the person giving the instruction. This is referred to as functional independent authority (Overheid.nl 1993).

Similar regulations apply to Belgium, with the only exception of isotonic fluids; Belgian nurses are allowed to start, change or stop these fluids without a prescription or order. A survey study by Wuyts et al. (2022) revealed that 56% of the Belgian nurses reported feeling a shared responsibility in fluid management. Additionally, Belgian nurses often intervened in urgent situations by choosing an intravenous fluid independently (Wuyts et al. 2022). In Scandinavia, nurses are allowed to determine the indication for administering intravenous fluids according to available guidelines (Baumgarten et al. 2019). The practices of nurses in the postanaesthesia care unit were explored, showing that the indications for fluid administration did not always fit with these guidelines (Baumgarten et al. 2019).

Once the current practices of Dutch nurses, expectations of Dutch prescribers, and the knowledge and documentation of both have been determined, it is possible to propose improvement strategies.

## 3 The Study

### 3.1 Aims

The primary aim of this study was to explore self‐reported practices and knowledge of nurses and prescribers regarding intravenous fluid therapy in general hospital wards. A secondary aim was to gain insights into how nurses and prescribers document intravenous fluid therapy in electronic healthcare charts.

## 4 Methods

### 4.1 Design

A multicentre cross‐sectional study comprising a survey study among healthcare professionals regarding intravenous fluid therapy, and a retrospective chart review to assess documentation of intravenous fluid therapy, conducted between April 2022 and July 2022.

### 4.2 Study Setting and Sampling

This study was carried out on 13 wards in four hospitals in the Southwest of the Netherlands, including one university hospital, two teaching hospitals and one general hospital.

### 4.3 Inclusion and Exclusion Criteria

The survey study targeted healthcare professionals such as nurses, nursing students, nurse practitioners, and physicians from surgical, non‐surgical and mixed wards, with each ward employing 30–100 nurses. The aim was to enrol at least 25 healthcare professionals per ward. Participants were divided into two groups: one of nurses and nursing students. Nursing students are allowed to administer and monitor intravenous fluids, under the indirect supervision of a nurse. Just like nurses, they are not allowed to start, change or stop without a prescription from a physician. Nursing students are an important part of the future healthcare workforce. Therefore, we were also curious about their knowledge and views. The other group consists of healthcare professionals licensed to prescribe intravenous fluid therapy, including physicians and nurse practitioners. For the remainder of this paper, these groups will be referred to as ‘nurses’ and ‘prescribers’, respectively. Recruitment methods included posters, emails and face‐to‐face invitations, performed by baccalaureate nursing students.

The same baccalaureate nursing students collected data from conveniently selected electronic healthcare charts of patients on the corresponding wards. All patients admitted for at least 48 h were eligible for inclusion. We aimed to include approximately 25 patients per ward.

### 4.4 Instruments

For the survey study, a literature search yielded a few questionnaires that covered knowledge of intravenous fluid therapy (Njung'e and Kamolo 2021; Sindahl et al. 2021; Tailor et al. 2020). However, most were either context‐specific or the questionnaire was not accessible. Therefore, baccalaureate nursing students under the expert supervision of an internist (J.A.) and a cardiac care nurse (D.S.) developed a Dutch questionnaire based on their experience and a review of the literature, translated to English for the purpose of this paper (see Data S1). The questionnaire opened with a statement to clarify that this questionnaire consisted of questions regarding intravenous fluids without the addition of medication, namely, glucose 5%, sodium chloride 0.9%, Ringer's solution, glucose 2.5%/sodium chloride 0.45%, gelofusine, mannitol and voluven. The questionnaire consisted of two parts and collected respondents' background characteristics. Respondents' characteristics included profession, ward, and years of work experience. The nursing students were also asked to fill in their years of experience; they indicated their years of experience as zero, with two exceptions. First, if the nursing student had previous experience as a nurse; there are two levels of nursing education in the Netherlands, vocational and baccalaureate. If a nursing student already finished their vocational study, they could count these years as years of experience as well. Second, student nurses that were employed in the hospital as a student nurse they could indicate their time as intern on the wards as well. Nurses were asked if they had completed a specialised training such as Oncology care or the Basic Intensive Acute Care (BIAZ) course. The BIAZ course trains in recognising abnormalities in vital signs, electrolyte disturbances, and how to effectively intervene.

The first part dealt with self‐reported practices in terms of nurses' initiative in starting, stopping or changing intravenous fluids and prescribers' expectations' regarding nurses taking initiative. The second section concerned knowledge of indications, contra‐indications and complications of intravenous fluid therapy. All knowledge questions were multiple‐choice, with at least three answer options. This questionnaire was piloted by baccalaureate nursing students and, due to multiple correct answers or interpretability issues, was subsequently slightly adjusted with the research group (J.A., D.S., M.D., E.I.). Eventually, 12 knowledge questions were selected.

**FIGURE 1:**
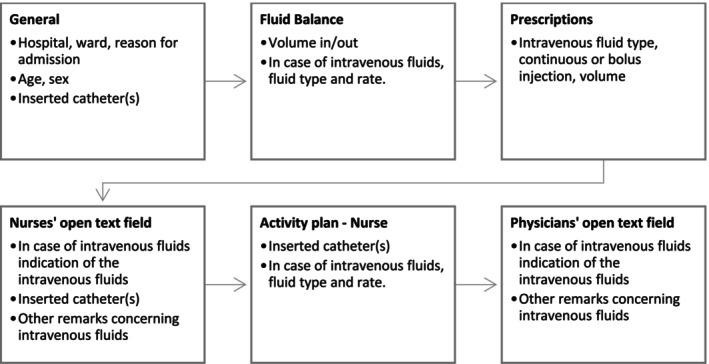
Structure of the chart review checklist: Indicating which data was collected from which section of the electronic patient file.

Data for the chart review were collected with a predefined checklist, structured to match the sections in the electronic patient records (Figure 1). The data was collected for the previous 24 h of admission, and included reason for admission, preconditions, age and sex, along with documentation of administered intravenous fluids. In case of intravenous fluid administration, the administered fluid type (e.g., sodium chloride 0.9%, Ringers solution, dextrose 5%), fluid rate (in mL/h), duration of administration, and the nurses' and prescribers' open text fields. ‘Keep‐the‐vein‐open’ was defined as continuous intravenous fluid therapy administered at a rate of up to 30 mL/h (Paquet and Marchionni 2016).

### 4.5 Data Collection

The questionnaire was conducted using LimeSurvey GmbH (n.d.). Participation was anonymous and could be performed on all devices with an internet connection. All questions were mandatory, and participants who did not complete all questions were excluded from the study.

For the chart reviews, the first author trained the baccalaureate nursing students in the data collection process during a 1‐h video call, in which the predefined checklist was walked through entirely with a fictive patient. The checklist was integrated in LimeSurvey. All hospitals used the same electronic patient file software (Chipsoft), which ensured a structured data collection process (Figure 1).

### 4.6 Data Analysis

Participant characteristics and multiple‐choice questions were analysed using descriptive statistics. Differences in responses between groups for each knowledge question were assessed with the chi‐squared test or Fisher's exact test, as appropriate. Some knowledge questions had open‐ended answers, which were categorised and rated for relevance by two researchers (D.S. and M.V.D.Z.). Quotes were selected to complement the results from the questionnaire.

The results from the chart review were analysed using descriptive statistics. The structure of the chart review prepared for student to identify any missing values, for instance when the question ‘is this patient receiving intravenous fluids’ was answered with yes, additional questions about the type of fluid and the infusion rate were questioned. If there was no description the value was indicated as missing. No missing data were imputed.

The data from the questionnaire and the chart review were analysed using IBM SPSS Statistics for Windows (version 28.0; IBM Corp., Armonk, NY). *p* < 0.05 were considered statistically significant.

### 4.7 Ethical Considerations

The Medical Ethics Review Committee of the Erasmus University Medical Center assessed this study as not subject to the Dutch Medical Research Involving Human Subjects Act and confirmed that gathering informed consent was not necessary (MEC‐2021‐0133).

## 5 Results

### 5.1 Demographics of the Participants of the Survey

Of the 427 participants who started the questionnaire, 304 (304/427; 71%) completed it. The majority of participants were nurses (279/304; 92%). See Table 1 for the baseline characteristics of the participants who completed the questionnaire. There was no significant difference between those who completed the questionnaire and those who did not in terms of age (median age for non‐completers: 25 years; IQR 22–36) and work experience in years (median work experience for non‐completers: 2 years; IQR 1–5).

**TABLE 1:** Nurses and prescribers demographics.

	Nurses (%)	Prescribers (%)	All participants (%)
Participants	279 (91.7)	25 (8.2)	304 (100)
Nursing students	36 (12.9)	—	36 (11.8)
BIAZ	40 (14.3)	1 (4.0)	41 (13.5)
Years of work experience	3 [2–8]	3 [1–5]	3 [2–8]
Age	27 [23–37]	33.0 [27.5–40.5]	28 [23–37.5]
Hospital			
Teaching hospital	178 (63.8)	14 (56.0)	192 (63.2)
University hospital	87 (31.2)	9 (36.0)	96 (31.6)
General hospital	14 (5.0)	2 (8.0)	16 (5.2)
Ward			
Surgical	124 (44.4)	7 (28.0)	131 (43.1)
Non‐surgical	84 (30.1)	12 (48.0)	96 (31.6)
Mixed	71 (25.4)	6 (24.0)	77 (25.3)

*Note:* Additional training for nurses to recognise abnormalities in vital signs, electrolyte disturbances, and how to effectively intervene.

Abbreviation: BIAZ, Basic Intensive Acute Care certificate.

Nearly two‐thirds of the participants (192/304; 63%) worked in one of the two teaching hospitals, 96 participants (96/304; 32%) in the academic hospital and 16 (16/304; 5%) in the general hospital. The median work experience was 3 years (IQR 2–8 years). Almost half of the registered nurses (105/243; 43%) reported insufficient knowledge gained during their initial training on intravenous fluid therapy, compared to 24% (6/25) of the prescribers. Details of the participants' demographics and professional backgrounds are shown in Table 1.

### 5.2 Nurses' Self‐Reported Practices and Prescribers' Expectations

Nurses reported that they stop (206/279; 74%) or start (192/279; 69%) the intravenous fluid therapy or change the rate (192/279; 69%) on their own initiative. Almost 30% (82/279; 29%) stated that they change the type of fluid without consulting a physician. The majority of the nurses (216/279; 77%) elaborated on reasons to start, stop or change the intravenous fluid therapy. Among those, 29 (29/216; 13%) nurses emphasised that they discussed their actions with the prescriber immediately after changing the infusion rate or type of fluid. A postoperative switch from Ringers lactate to sodium chloride 0.9% solution was commonly mentioned.

*Nurse 185*: If a patient suddenly has a fever, or suddenly has to start fasting, and the doctor has not yet assessed the patient, and the patient needs more infusion for those reasons. Also, if I suspect that a patient is decompensated and I have called the doctor about this and I am still waiting for the doctor's assessment, I often temporarily lower the infusion.

*Nurse 347*: When the patient drinks sufficiently I lower the infusion.

*Nurse 93*: Guided by patient intake, patient history.

Most of the prescribers (20/25; 80%) expect that nurses independently start, stop or change intravenous fluid therapy, commenting that they trust the nurses' expertise in most cases, but expect to be consulted in more complex situations.

*Prescriber 465*: In most cases, I believe ward nurses can make a good assessment of whether there is sufficient intake and when the infusion can be phased‐out and/or discontinued. I think nurses can make a good assessment when to start infusion if there is a lot of fluid loss and little intake. Adjusting the infusion, especially major adjustments, is often preferable in consultation, given some patients' histories or disease progression and in order to maintain a little more control and overview.

*Prescriber 399*: The nurses on the ward have a lot of experience with infusion therapy and know how to handle it well, when in doubt they consult with the physician on duty.

### 5.3 Knowledge of Nurses' and Prescribers Concerning Intravenous Fluid Therapy

Overall, the median number of correct answers was 8 out of 12 questions (IQR 7–9, range 0–12). Four participants (4/304; 1.3%), all with a nursing background, answered all knowledge questions correctly. One of the participants, a registered nurse, answered none of the questions correctly. Most participants answered the questions about indications, contraindications, complications, and symptoms of decompensation of intravenous fluid therapy correctly. There were no significant differences between nurses and prescribers for most questions, except for the question on the indications of intravenous fluids (*p* = 0.042).

There was only one participating ward from the general hospital, making statistical comparisons with the teaching hospitals or the university hospital unjustifiable (respectively 14, 178 and 87 nurses, and respectively 2, 14 and 9 prescribers). Overall, the median number of correctly answered questions at the general hospital was lower for the nurses and higher for the prescribers. In contrast, there was no difference in median scores (eight correct answers) between nurses and prescribers in the teaching and university hospital. Table 2 presents the results of the knowledge questions per participant group.

**TABLE 2:** Knowledge questions and answers, overall by nurses and prescribers and per type of hospital.

Questions^a^	All nurses % (*N* = 279)	Nurses teaching hospitals % (*n* = 178)	Nurses university hospital % (*n* = 87)	Nurses general hospital % (*n* = 14)	All prescribers % (*N* = 25)	Prescribers teaching hospitals % (*n* = 14)	Prescribers university hospital % (*n* = 9)	Prescribers general hospital % (*n* = 2)	*p*
Overall correct answered questions (median, IQR)	8 [7–9]	8 [7–9]	8 [7–9]	6.5 [6–7.25]	8 [7–10]	8 [7–10]	8 [7–9.5]	9.5 [NA]	
1. What is *NOT* an indication for intravenous fluid(s) therapy?									1
Preoperative intravenous fluid therapy of 21 mL/h with a eGFR of 90	69.5 (194)	70.8 (126)	65.5 (57)	78.6 (11)	72.0 (18)	71.4 (10)	66.7 (6)	100 (2)	
b Dehydration after losing 2 L of blood perioperative	3.9 (11)	2.8 (5)	6.9 (6)	0	8.0 (2)	7.1 (1)	11.1 (1)	0	
c Patient who switched from normal diet to NPO (nil‐by‐mouth)	26.5 (74)	26.4 (47)	27.6 (24)	21.4 (3)	20.0 (5)	21.4 (3)	22.2 (2)	0	
2. What *IS* an indication for intravenous fluid(s) therapy?									0.042*
When a patient has a negative fluid balance on account of prolonged vomiting	92.8 (259)	96.6 (172)	83.9 (73)	100 (14)	80.0 (20)	78.6 (11)	77.8 (7)	100 (2)	
b When intravenous antibiotics are prescribed	3.6 (10)	1.7 (3)	8.0 (7)	0	8.0 (2)	7.1 (1)	11.1 (1)	0	
c When intravenous fluid(s) therapy is giving to keep‐the‐vein‐open	3.6 (10)	1.7 (3)	8.0 (7)	0	12.0 (3)	14.3 (2)	11.1 (1)	0	
3. What are symptoms of fluid overload?									0.650
Shortness of breath, edema, high blood pressure, decreased appetite	94.5 (263)	94.9 (169)	83.1 (81)	92.9 (13)	92.0 (23)	100 (14)	77.8 (7)	100 (2)	
b Shortness of breath, sweatiness, low blood pressure	2.2 (6)	0.6 (1)	4.6 (4)	7.1 (1)	0	0	0	0	
c Feeling weak, cramps, dizziness, low blood pressure	3.6 (10)	4.5 (8)	2.3 (2)	0	8.0 (2)	0	22.2 (2)	0	
4. What complications may occur when administering intravenous fluid(s) therapy?									0.146
Fever, decompensation, shortness of breath	90.3 (252)	92.1 (164)	88.5 (77)	78.6 (11)	100 (25)	100 (14)	100 (9)	100 (2)	
b Dizziness, skin tear, coughing	2.2 (6)	1.1 (2)	3.4 (3)	7.1 (1)	0	0	0	0	
c Dehydration, coughing, shortness of breath	7.5 (21)	6.7 (12)	8.0 (7)	14.3 (2)	0	0	0	0	
5. In which of the following situations is it recommended to give glucose 5% intravenous fluid?									0.093
A patient with a good kidney function with hypernatremia and NPO status	44.8 (125)	41.0 (73)	58.6 (51)	7.1 (1)	64.0 (16)	64.3 (9)	55.6 (5)	100 (2)	
b A patient known to have diabetes mellitus type 2 who is unconscious with a blood sugar level of 2.1 mmol/L	48.7 (136)	53.9 (96)	31.0 (27)	92.9 (13)	36.0 (9)	35.7 (5)	44.4 (4)	0	
c A patient with hypovolemia	6.5 (18)	5.1 (9)	1.3 (9)	0	0	0	0	0	
6. In which of the following situations is it recommended to give lactated Ringer's solution?									0.263
A patient who is intravascularly dehydrated, hypotensive, and has a NPO status	67.7 (189)	69.7 (124)	63.2 (55)	71.4 (10)	80.0 (20)	71.4 (10)	88.9 (8)	100 (2)	
b If sodium chloride 0.9% is not available, and speed is required	14.3 (40)	14.0 (25)	61.1 (14)	7.1 (1)	16.0 (4)	28.6 (4)	0	0	
c A patient with a potassium level of 4.0 mmol/L	17.9 (50)	16.3 (29)	20.7 (18)	21.4 (3)	4.0 (1)	0	11.1 (1)	0	
7. The physician asks you to get glucose for a patient with severe hypoglycemia; you see glucose 5%, 10% and 20%, which one do you take?									0.888^&^
20% glucose solution	57.7 (161)	52.2 (93)	67.8 (59)	64.3 (9)	56.0 (14)	64.3 (9)	33.3 (3)	100 (2)	
b 5% glucose solution	22.2 (62)	24.7 (44)	16.1 (14)	28.6 (4)	20.0 (5)	7.1 (1)	44.4 (4)	0	
c 10% glucose solution	20.1 (56)	23.0 (41)	16.1 (14)	7.1 (1)	24.0 (6)	28.6 (4)	22.2 (2)	0	
8. Which action suits best for a patient who is responsive and allowed to eat and drink normally, with a hypokalemia of 2.8?									0.669
Potassium drink, if the patient is able to drink	62.4 (174)	62.4 (111)	69.0 (60)	21.4 (3)	68.0 (17)	71.4 (10)	66.7 (6)	50 (1)	
b Start with a potassium infusion in combination with sodium chloride 0.9% solution	35.8 (100)	34.8 (62)	31.0 (27)	78.6 (11)	32.0 (8)	28.6 (4)	33.3 (3)	50 (1)	
c Start with lactated Ringer's solution	1.8 (5)	2.8 (5)	0	0	0	0	0	0	
9. The daily reference intake of salt is 6 g per day. For cardiac patients and patients witch renal failure on a low‐sodium diet, the reference intake is lower. *How much salt contains 1 L of sodium chloride 0.9%?*									0.094
9 g	41.9 (117)	41.0 (73)	47.1 (41)	21.4 (3)	60.0 (15)	57.1 (8)	55.6 (5)	100 (2)	
b 0.9 g	40.5 (113)	42.1 (75)	35.6 (31)	50.0 (7)	20.0 (5)	21.4 (3)	22.2 (2)	0	
c 9 mg	17.6 (49)	16.9 (30)	17.2 (15)	28.6 (4)	20.0 (5)	21.4 (3)	22.2 (2)	0	
10. What are possible symptoms of hypernatremia?									0.090
Thirst, confusion, neurological deficit, nausea	88.2 (246)	88.2 (157)	88.5 (77)	85.7 (12)	100 (25)	100 (14)	100 (9)	100 (2)	
b Diarrhoea, dyspnoea, white skin colour	5.7 (16)	7.3 (13)	3.4 (3)	0	0	0	0	0	
c Loss of appetite, losing weight, lethargy	6.1 (17)	4.5 (8)	8.0 (7)	14.3 (2)	0	0	0	0	
11. What infusion fluid is preferred for dialysis patients?									0.531
Lactated Ringer's solution	58.8 (164)	54.5 (97)	77.8 (59)	57.1 (8)	52.0 (13)	57.1 (8)	55.6 (5)	0	
b Sodium chloride 0.9%	17.9 (50)	18.5 (33)	12.6 (11)	42.9 (6)	28.0 (7)	28.6 (4)	22.2 (2)	50 (1)	
c No preference	23.3 (65)	27.0 (48)	19.5 (17)	0	20.0 (5)	14.3 (2)	22.2 (2)	50 (1)	
12. A patient's planned surgery is postponed to 15:00, current time is 11:00. The patient receives a keep‐the‐vein‐open infusion, and has been fasting since midnight. What do you think is the best intervention?									0.436
Give a glass of water up to 2 h before surgery	19.7 (55)	24.7 (44)	11.5 (10)	7.1 (1)	12.0 (3)	7.1 (1)	22.2 (2)	0	
b Do not change the infusion rate	19.0 (53)	22.5 (40)	13.8 (12)	7.1 (1)	32.0 (8)	42.9 (6)	22.2 (2)	0	
c Increase the infusion rate to prevent dehydration	59.1 (165)	50.6 (90)	72.4 (63)	95.7 (12)	56.0 (14)	50.0 (7)	55.6 (5)	100 (2)	
d Stop the infusion to prevent decompensation	2.2 (6)	22.2 (4)	2.3 (2)	0	0	0	0	0	

*Note:* All statistical tests were performed to assess the difference between all nurses and all prescribers. All tests had to be performed with the two‐sided Fisher's exact test (good vs. wrong answers), except for the one indicated with ‘&’, which was performed with the chi‐squared test (all answers). ‘a.’ indicates the correct answer, this was not the order of the answers in the actual questionnaire, and the order is shown in Data S1.

^a^ All questions are about the intravenous fluid therapy without addition of medication. This concerns intravenous fluids such as glucose 5%, sodium chloride 0.9%, lactated Ringer's solution, glucose 2.5%/sodium chloride 0.45%, gelofusine, mannitol and voluven.

Twenty percent (55/279) of the nurses, and 12% (3/25) of the prescribers indicated compliance with the current fasting guideline in case of postponed surgery. More than half of the participants (165/279; 59% nurses; 14/25; 56% prescribers) preferred to increase the infusion rate (see question 12). Question 9, which assessed knowledge of the amount of salt in 1 L of sodium chloride 0.9% solution, was answered incorrectly by 58% of the nurses (162/279) and 40% of the prescribers (10/25). The correct indication for administering glucose 5% was identified by 45% of the nurses (125/279) and 64% of the prescribers (16/25) (Table 2).

### 5.4 Patients Characteristics From the Chart Review

The charts of 362 patients were screened: 252 from the teaching hospitals, 86 from the university hospital and 25 from the general hospital. The median age of these patients was 69 years (IQR 58–77); 47% (169/362) were female. Most of them (329/362; 91%) had an intravenous catheter in situ, most commonly a peripheral catheter (295/329; 90%). Table 3 presents the patient characteristics.

**TABLE 3:** Characteristics of the patients from the chart reviews.

	Teaching hospitals	University hospital	General hospital	All (%)
Patients (*n*, %)	251 (69.3)	86 (23.8)	25 (6.9)	362 (100)
Age (median [IQR])	70 [58–78]	65 [50–72]	75 [68.5–84.5]	69 [58–77]
Sex (female, %)	122 (48.6)	33 (38.4)	14 (56.0)	169 (46.7)
Ward				
Surgical	100 (39.7)	50 (57.5)	0	150 (41.4)
Mixed	49 (19.4)	36 (42.5)	25 (100)	110 (30.4)
Non‐surgical	102 (40.5)	0	0	102 (28.2)
Intravenous access in situ				
Kind of access not registered	5 (2.0)	8 (9.3)	3 (12.0)	16 (4.4)
PVC	221 (88.0)	56 (65.1)	18 (72.0)	295 (81.5)
CVC	4 (1.6)	2 (2.3)	0	6 (1.7)
PICC	6 (2.4)	5 (5.8)	1 (4.0)	12 (3.3)
No access	15 (6.0)	15 (17.4)	3 (12.0)	33 (9.1)
Intravenous fluids administered during the previous 24 h	135 (53.8)	51 (59.3)	10 (40.0)	196 (54.1)
Continuous	133 (53.0)	48 (55.8)	10 (40.0)	191 (52.8)
Bolus injection	6 (2.4)	3 (3.5)	0	9 (2.5)
Type of fluid				
Sodium chloride 0.9%^a^	119 (88.1)	41 (80.4)	8 (80.0)	168 (85.7)
Ringer's lactate^a^	9 (6.7)	2 (3.9)	2 (20.0)	13 (6.6)
Unknown^a^	7 (5.2)	8 (15.7)	0	15 (7.7)
Keep‐the‐vein‐open				
Fluid rate < 30 ml/h^a^	58 (43.0)	17 (33.3)	2 (20.0)	77 (39.3)
Fluid rate 42 mL/h (1000 mL/24 h)^a^	17 (12.6)	8 (15.7)	4 (40.0)	29 (14.8)
Fluid rate 63 mL/h (1500 mL/24 h)^a^	18 (13.3)	8 (15.7)	0	26 (13.3)
Fluid rate 84 mL/h (2000 mL/24 h)^a^	22 (16.3)	14 (27.5)	2 (20.0)	38 (19.4)
Fluid rate 126 mL/h (3000 mL/24 h)^a^	3 (2.2)	1 (2.0)	1 (10.0)	5 (2.6)
Unknown fluid rate^a^	17 (12.6)	3 (5.9)	1 (10.0)	21 (10.7)

Abbreviations: CVC, central venous catheter; PICC, peripherally inserted central catheter; PVC, peripheral venous catheter.

^a^ Percentages calculated from ‘Intravenous fluids administered during the previous 24 h’.

### 5.5 Documentation of Intravenous Fluid Therapy

Intravenous fluid therapy entries were found in multiple sections of the electronic patient files. Nearly two‐thirds (196/329; 60%) of the patients with intravenous access received intravenous fluids during the previous 24 h of admission. In 97% of cases (190/196), the prescribers did not document the indication for intravenous fluid therapy. Conversely, nurses documented the indication in 40 cases (40/196; 20%).

The patients that received intravenous fluids received them continuously (191/196; 97%), while nine (9/196; 5%) received an additional bolus injection. Most patients (168/195; 86%) received sodium chloride 0.9%, followed by Ringer's solution (13/195; 7%). In 15 cases (15/195; 8%) the type of fluid was not documented. In accordance with our definition of ‘to keep‐the‐vein‐open’ with a maximum fluid rate of 30 mL/h, 77 (77/196; 40%) patients received intravenous fluids to keep‐the‐vein‐open. However, 21 charts (21/196; 11%) did not specify the infusion rate. The infusion rates over 30 mL/h were all multiplications of 500 mL (Table 3). The infusion rates were documented contradictorily within the different sections of the charts for 18 patients' charts (18/196; 9%); the type of fluid was documented contradictorily between different sections in 10 patients' charts (10/196; 5%).

## 6 Discussion

### 6.1 Summary of Key Findings

This study explored nurses' and prescribers' practices and knowledge regarding intravenous fluid therapy on general hospital wards in the Netherlands. Our study found that prescribers generally expect nurses to intervene when necessary, and that nurses often met these expectations by independently adjusting intravenous fluid therapy. Both groups demonstrated knowledge gaps, with participants answering 4 out of 12 knowledge questions incorrectly on average. These gaps included limited awareness of the composition of 0.9% sodium chloride and uncertainty about the appropriate glucose concentration for treating severe hypoglycaemia. Patient chart reviews indicated that 90% of patients receiving intravenous fluids were administered 0.9% sodium chloride, typically in multiplications of 500 mL, which may increase the risk of excessive sodium intake. Additionally, our findings suggest that the frequent use of keep‐the‐vein‐open rates and the infrequent documentation of intravenous fluid therapy indications could hinder clinicians' ability to evaluate the ongoing appropriateness and effectiveness of intravenous fluids.

### 6.2 Responsibility and Knowledge of Intravenous Fluid Therapy

Knowledge gaps concerning intravenous fluids have been reported in various countries and clinical settings. For instance, Nasa et al. (2022) performed an international cross‐sectional survey in perioperative and critical care settings and found a considerable variation in knowledge among healthcare professionals, with higher scores among supervisors, specialists and professionals in high‐income countries (Nasa et al. 2022). In our study, participants answered 8 out of 12 knowledge questions correctly on average, indicating that knowledge gaps persist—particularly regarding nil‐by‐mouth policies and the composition or indication of commonly used intravenous fluids. These gaps, combined with limited documentation of the indications for intravenous fluids, may reflect a lack of awareness among participants about the importance of adequate knowledge and potential risks associated with intravenous fluid administration.

Although nurses from general hospital wards are not formally responsible for prescribing intravenous fluids, our findings suggest that many nurses independently start, stop or change intravenous fluid therapy. This aligns with findings by Eastwood et al. (2015), who reported that nurses from Intensive Care wards actively contribute to intravenous fluid therapy decisions during daily rounds or in non‐urgent situations. However, most nurses in our study emphasised that independent adjustments typically occur in acute situations or while awaiting a physician's assessment, which may reflect differences in physician availability between intensive care and general ward settings—particularly during evenings, nights and weekends.

Previous studies have shown that nurse‐led protocols in specialised care wards can reduce unnecessary intravenous fluid therapy (Brien et al. 2020; Fahlstrom et al. 2013). These protocols help ensure that fluid therapy is regularly evaluated and adjusted, rather than relying on daily rounds with prescribers. Similarly, Makaryus et al. (2018) proposed a visual general guideline for emergency room patients to minimise preoperative nil‐by‐mouth time and discontinue intravenous fluids postoperatively. While promising, such guidelines should be adapted to general ward settings and integrated with considerations such as fluid and electrolyte balance (NICE 2017), blood pressure (NICE 2017; Penmatsa et al. 2021), and other clinical factors.

Despite existing regulations in the Netherlands, indicating that nurses need formal instruction from an independently authorised healthcare provider to start, change or stop intravenous fluids, our study found that prescribers often expect nurses to independently adjust the intravenous fluid policy. This perceived shared responsibility suggests a need to revisit current policies and formally recognise intravenous fluid therapy as a collaborative task between prescribers and nurses. Such recognition could support the development of joint protocols aimed at improving the safety and effectiveness of intravenous fluid therapy on general hospital wards.

### 6.3 Targeted Intravenous Fluid Therapy

As with all treatments, intravenous fluid therapy should only be administered when clinically indicated. The three primary indications for intravenous fluid therapy are resuscitation, replacement and maintenance (Malbrain et al. 2020; Ramsay et al. 2018). Ideally, intravenous therapy prescriptions should be tailored to patient needs, particularly based on these indications (NICE 2017). This approach is standard in paediatric care, where fluid volumes are routinely calculated per kilogram of body weight (Morice et al. 2022). However, our study found limited evidence of such tailoring. Nearly all documented intravenous fluid infusion rates in the patients' chart were standardised volumes—typically multiplications of 500 mL—with the only deviations being low rates (5 and 10 mL/h) used to keep‐the‐vein‐open. This pattern suggests that intravenous fluid therapy was not according to the three primary indications for intravenous fluid therapy. This raises the possibility that some patients may have received suboptimal fluid volumes—either insufficient or excessive. Furthermore, previous research had highlighted concerns in prescribing practices (Gao et al. 2015). For instance, Sneyers et al. (2025) reported that 60% of the patients who received intravenous fluids were inappropriate, representing an excessive median volume of 300 mL per patient, and median costs of €4.60 per patient. This raises questions about the patient centredness of care and lack of awareness concerning resource management and sustainability in health care.

### 6.4 Low‐Value Care

Low‐value care—defined as the overuse or inappropriate use of treatments and medical tests—remains a persistent issue across many Western healthcare systems (Brownlee et al. 2017). Despite increasing awareness of the harms of low‐value care, such practices persist, with notable variation across regions and healthcare settings (Segal et al. 2022). Our study identified an example of potentially low‐value care related to intravenous fluid therapy. Particularly, given the fact that in the context approximately one‐third of patients received intravenous fluids at rates below 30 mL/h—suggesting the use of keep‐the‐vein‐open infusions. This practice may represent overuse, particularly given evidence that a saline lock, flushed once per 24 h, is equally effective, less costly, and associated with fewer risks than using continuous intravenous fluids to keep the vein open (Roszell et al. 2018; Schreiber et al. 2015).

In addition to clinical complications such as electrolyte imbalances, reduced mobility and patient discomfort (Wesselius et al. 2018), unnecessary intravenous fluid administration contributes to inappropriate care. Unnecessary healthcare interventions, increased costs, and material waste have a major impact on the environmental footprint of healthcare (Leal Filho et al. 2024). This sector is responsible for approximately 5% of global greenhouse gas emissions (Karliner et al. 2020).

However, a limitation is that our study did not account for clinical exceptions where continuous intravenous fluids may be indicated. For example, frequent intravenous medication administration may justify the use of a keep‐the‐vein‐open rate to reduce the need for repeated line connections and disconnections, which can increase infection risk. According to infection prevention guidelines, intravenous lines should be discarded after each disconnection to prevent contamination (Guidelines Review Committee Infection Prevention and Control [IPC] 2024), a practice that may not be feasible with frequent intermittent medication administration (Hunfeld et al. 2023; United Nations 2023).

Another potentially low‐value care practice is preoperative intravenous fluid use (Brady et al. 2003). Specifically, 80% of nurses and 88% of prescribers in our sample did not adhere to established preoperative fasting guidelines for patients with nil‐by‐mouth advice. This finding is consistent with van Noort et al. (2021), who reported about fasting habits over a 10‐year period. They found that nil‐by‐mouth guidelines were incorrectly followed in over 90% of the cases, often resulting in unnecessarily prolonged fasting for both solid foods and clear liquids (van Noort et al. 2021).

Furthermore, rethinking the indication of peripheral intravenous catheters could lead to more appropriate care. For many patients, having a peripheral intravenous catheter inserted is not just a minor procedure—it can be painful, stressful and emotional (Cooke et al. 2018). On top of that, it is not uncommon for patients to feel sidelined in decisions about whether the peripheral intravenous catheter is truly necessary or how it is managed (Cooke et al. 2018). Research has shown that these devices are often used inappropriately or without a clear clinical justification (Laan et al. 2020). Rethinking their routine use—not just from a patient‐centered perspective, but also in terms of reducing unnecessary fluid therapy—could have meaningful environmental benefits as well (Touw et al. 2023).

Taken together, our findings underscore the need for greater adherence to evidence‐based guidelines and a more critical evaluation of routine practices surrounding intravenous fluid therapy, particularly in non‐urgent and perioperative contexts.

### 6.5 Strengths and Limitations

This study was conducted across multiple hospital wards, and the consistency of findings across wards and hospitals suggests that the results may be transferable to other general hospital settings in the Netherlands. However, the sample was predominantly composed of nurses (92%), with relatively few prescribers participating. While this reflects the staffing ratios on general wards—where nurse‐to‐patient ratios are approximately 1:4–6 and prescriber‐to‐patient ratios range from 1:5–30—it may limit the generalizability of findings related to prescriber practices.

There is a potential risk for selection bias, as participants may have been those with a particular interest in intravenous fluid therapy, possibly underrepresenting those who do not prioritise this aspect of care. To mitigate this, we recruited participants through nursing students on their own wards and ensured anonymity to encourage broad participation. However, due to the pragmatic and varied recruitment methods, we were unable to calculate an exact response rate.

Furthermore, one specific limitation relates to question 12 in the knowledge survey, which asked participants to respond to a scenario involving a postponed surgery. In retrospect, it would have been valuable to explore participants' reasoning and their familiarity with current preoperative fasting guidelines to better interpret their responses.

Although triangulating data from the knowledge survey and patient chart reviews on the same hospital wards provided valuable insights, the use of a 12‐question knowledge survey remains a limitation. While pragmatic and time‐efficient, it may have limited the depth of clinical context captured and allowed for varied interpretation. Additionally, reliance on retrospective clinical documentation introduces potential biases, including incomplete records and limited insight into the clinical reasoning behind decisions. These methodological constraints should be considered when interpreting our findings.

## 7 Conclusions

Our study identified a clear knowledge gap among healthcare professionals regarding intravenous fluid therapy. We also observed limited documentation of the clinical indication for intravenous fluid use and frequent reliance on keep‐the‐vein‐open rates. While intravenous fluids are commonly administered during hospitalisation, our findings suggest that not all patients may require them and that current practices may not always reflect individualised, evidence‐based care. The combination of knowledge deficits and insufficient documentation points to a broader lack of awareness about the risks and responsibilities associated with intravenous fluid therapy among healthcare providers. This suggests that intravenous fluids may not always be approached with the same clinical rigour as other medications. Importantly, the perceived shared responsibility between nurses and prescribers presents an opportunity. Recognising intravenous fluid therapy as a collaborative task could serve as a foundation for developing joint protocols that support more targeted, appropriate, and safe fluid management on general hospital wards.

## Author Contributions

D.S., E.I., M.Z., M.D., E.B., C.U. and J.A.: Made substantial contributions to conception and design or acquisition of data or analysis and interpretation of data. D.S., E.I., M.Z., M.D. and J.A.: Involved in drafting the manuscript or revising it critically for important intellectual content. D.S., E.I., M.Z., M.D., E.B., C.U. and J.A.: Gave final approval of the version to be published. And agreed to be accountable for all aspects of the work in ensuring that questions related to the accuracy or integrity of any part of the work are appropriately investigated and resolved.

## Disclosure

There is a statistician on the author team; Monique van Dijk. *Trial and protocol registration*: No trial or protocol registration was deemed necessary due to the design (knowledge questionnaire and retrospective review).

## Ethics Statement

The Medical Ethics Review Committee of the Erasmus University Medical Center assessed this study as not subject to the Medical Research Involving Human Subjects Act (MEC‐2021‐0133).

## Conflicts of Interest

The authors declare no conflicts of interest.

## Supporting information


**Data S1:** jan70216‐sup‐0001‐DataS1.docx.

**Data S2:** jan70216‐sup‐0002‐DataS2.docx.

## Data Availability

The data that support the findings of this study are available from the corresponding author upon reasonable request.
