# Supplementary material for: Nurses' Self‐Reported Practices and Prescribers' Expectations in Intravenous Fluid Therapy for Hospitalised Patients: A Survey Study and Clinical Documentation Review
Source: J Adv Nurs. 2025 Sep 11;82(6):5951–66. doi: 10.1111/jan.70216 (PMC13176734; doi:10.1111/jan.70216)
Supplement: Supplementary file 1 — Data S1: jan70216‐sup‐0001‐DataS1.docx. [file JAN-82-5951-s001.docx]

### Supplement 1 : Self-reported practice and knowledge questionnaire

Part 1 – self-reported practice about intravenous fluid therapy.

All questions are about the intravenous fluid therapy WITHOUT addition of medication. Therefore, this concerns intravenous fluids, namely Glucose 5%, Sodium Chloride 0.9%, Ringer’s solution, Glucose 2.5%/Sodium Chloride 0.45%, Gelofusine, Mannitol, Voluven.

| **Nurses and prescribers** | **Nurses** |
| --- | --- |
| 1. Did you visit an education class about intravenous fluid therapy?    - Yes    - No 2. Did you visit a congress/education day about intravenous fluid therapy?    - Yes    - No 3. What values do you look at before making a change?    - Vital signs (such as blood pressure)    - Clinical experience    - Medical history    - Laboratory values    - Other: ……. (open answer) 4. What else did you want to learn in your **primary education**?  - Fluid and electrolyte balance by healthy adults - The influence of surgery on fluid balance - Patient characteristics that are important to assess - Patient characteristics that may indicate fluid loss - Treatment of normal/too little/too much moisture - The electrolyte balance of the different available intravenous fluids - When you choose for which fluid - Other: ……. (open answer)  1. Did you gain enough knowledge about intravenous fluid therapy during **primary education**?    - Yes    - No 2. What is the keep-vein-open infusion rate according to you?  - 2-10 ml/hour - 10-21 ml/hour - >21 ml/hour - I am not in favor of keeping the vein open with continuous infusion  1. No right or wrong, preferred answer is bold: A patient with keep-the-vein-open infusion has NPO status since midnight because of a planned surgery or medical examination. What do you think it is the best intervention?  - Do not change the infusion rate - Increase the infusion rate to prevent dehydration - Stop the infusion to prevent decompensation - **Give a glass of water up to 2 hours before the operation/medical examination** | 1. Do you ever **change** the **rate** of a prescribed intravenous fluid therapy on your own initiative?    - Yes, namely…. (open answer)    - No 2. Do you ever **start** a fluid therapy on your own initiative? (Inclusive keep-the-vein-open)    - Yes, namely…. (open answer)    - No 3. Do you ever **stop** a fluid therapy on your own initiative? (Inclusive keep-the-vein-open)    - Yes, namely…. (open answer)    - No 4. Do you ever **change** the infusion **fluid** on your own initiative?    - Yes, namely…. (open answer)    - No 5. What are reasons for you to change the infusion fluid on your own initiative?    - Does not apply for me    - (long-term) nil-by-mouth    - Prevent dehydration    - Prevent fluid overload (decompensation cordis)    - Other: ……. (open answer) |
|  | **Prescribers** |
|  | 1. Do you (as a medical physician, nurse practitioner or physician assistant) expect nurses to start, stop or change the intravenous fluid therapy on own initiative?    - Yes    - No 2. Can you describe shortly what you expect from nurses about starting, stopping or changing the intravenous fluid therapy?   Open answer |

Part 2 - Knowledge questions

| **Questions for all participants** |  |
| --- | --- |
| 1.What is **NOT** an indication for intravenous fluid(s) therapy? | 7.The physician asks you to get glucose for a patient with severe hypoglycemia; you see glucose 5%, 10% and 20%, which one do you take? |
| **Preoperation intravenous fluid therapy of 21 ml/h with a eGFR of 90 (Normal > 90)** | 5% glucose solution |
| Dehydration after losing 2 liters of blood peri-operatively | 10% glucose solution |
| Patient who switched from normal diet to NPO (nil-by-mouth) | **20% glucose solution** |
| 2.What **IS** an indication for intravenous fluid(s) therapy? | 8.Which action suits best for a patient who is responsive and allowed to eat and drink normally, with a hypokalemia of 2.8? |
| When intravenous antibiotics are prescribed | **Potassium drink, if the patient is able to drink** |
| When intravenous fluid(s) therapy is giving to keep-the-vein-open | Start with a potassium infusion in combination with sodium chloride 0.9% solution |
| **When a patient has a negative fluid balance on account of prolonged vomiting** | Start with lactated Ringer’s solution |
| 3.What are symptoms of decompensation? | 9.The daily reference intake of salt is 6 grams per day. For cardiac patients and patients witch renal failure on a low-sodium diet, the reference intake is lower.  *How much salt contains 1 liter of sodium chloride 0.9%?* |
| Feeling weak, cramps, dizziness, low blood pressure | 0.9 grams |
| **Shortness of breath, edema, high blood pressure, decreased appetite** | **9 grams** |
| Shortness of breath, sweatiness, low blood pressure | 1. milligrams |
| 4.What complications may occur when administering intravenous fluid(s) therapy? | 10.What are possible symptoms of hypernatremia |
| **Fever, decompensation, shortness of breath** | Diarrhea, dyspnea, white skin color |
| Dizziness, skin tear, coughing | **Thirst, confusion, neurological deficit, nausea** |
| Dehydration, coughing, shortness of breath | Loss of appetite, losing weight, lethargy |
| 5.In which of the following situations is it recommended to administer glucose 5%? | 11.What infusion fluid is preferred for dialysis patients? |
| **A patient with a good kidney function with hypernatremia and NPO (nil-by-mouth) status** | Sodium chloride 0.9% |
| A patient known to have diabetes mellitus type 2 who is unconscious with a blood sugar level of 2.1 mmol/L | **Lactated Ringer’s solution** |
| A patient with hypovolemia | No preference |
| 6.In which of the following situations is it recommended to give lactated Ringer’s solution? | 12.A patient’s planned surgery is postponed to 15:00, current time is 11:00. The patient receives a keep-the-vein-open infusion, and has been fasting since midnight. What do you think is the best intervention? |
| If sodium chloride 0.9% is not available, and speed is required | Do not change the infusion rate |
| A patient with a potassium level of 4.0 mmol/L | Increase the infusion rate to prevent dehydration |
| **A patient who is intravascularly dehydrated, hypotensive, and has a NPO (nil-by-mouth) status** | Stop the infusion to prevent decompensation  **Give a glass of water up to 2 hours before the surgery** |
| The correct answers are shown in **bold.** | |
